# Analysis of Selected Cognitive Abilities and Temperamental Traits Among Young Adults Playing Video Games

**DOI:** 10.3390/brainsci16101109

**Published:** 2026-10-14

**Authors:** Justyna Krzepota, Krzysztof Wrześniewski, Joanna Basiaga-Pasternak, Marek Cieśluk, Aneta Łuć, Tomasz Sacewicz, Dorota Sadowska

**Affiliations:** 1 Institute of Physical Culture Sciences, University of Szczecin, 70-453 Szczecin, Poland; 2 Department of Psychology, Faculty of Physical Education and Sport, University of Physical Culture in Krakow, 31-571 Krakow, Poland; joanna.basiaga@awf.krakow.pl; 3 Department of Social and Developmental Psychology, Institute of Psychology, University of Szczecin, 70-453 Szczecin, Poland; marek.ciesluk@usz.edu.pl; 4 Regional Research and Development Center, Faculty of Physical Education and Sport in Biała Podlaska, Józef Piłsudski University of Physical Education in Warsaw, 21-500 Biała Podlaska, Poland; anetaluc1980@gmail.com; 5 Department of Biomechanics and Anatomy, Faculty of Physical Education and Health in Biała Podlaska, Józef Piłsudski University of Physical Education in Warsaw, 21-500 Biała Podlaska, Poland; tomasz.sacewicz@awf.edu.pl; 6 Institute of Sport—National Research Institute, 01-982 Warsaw, Poland; dorota.sadowska@insp.pl

**Keywords:** gamers, video games, cognitive abilities, Vienna Test System, temperamental traits, the Formal Characteristics of Behaviour—Temperament Inventory

## Abstract

**Objectives**: The aim of this study was to analyse cognitive functioning and temperamental traits among young adults differing in their level of engagement in video gaming. **Methods**: The analysis included 67 male students from the Józef Piłsudski University of Physical Education in Warsaw, Branch Campus in Biała Podlaska. Based on average daily gaming time, participants were classified as frequent players (n = 22; mean age 22.45 ± 2.40 years; 6.29 ± 6.07 h/day) or casual players (n = 45; mean age 21.60 ± 1.96 years; 0.94 ± 0.95 h/day). Cognitive functioning was assessed using the Reaction Test (RT), Signal Test (ST), and Determination Test (DT) from the Vienna Test System. Temperament was measured with the Formal Characteristics of Behaviour–Temperament Inventory. **Results**: Frequent players showed a trend toward shorter reaction time in RT/S3 (U = 356; *p* = 0.064). In the Signal Test, they demonstrated significantly fewer omitted reactions, more correct responses, higher focused attention, and better total results (all *p* < 0.01). Regarding temperament, frequent players scored significantly lower on briskness (*p* = 0.034) and rhythmicity (*p* = 0.021) than casual players. **Conclusions**: The present exploratory study suggests a nuanced relationship between media engagement, specific cognitive performance, and temperamental traits. Frequent players demonstrated a robust advantage in parameters of sustained attention, alongside a marginal descriptive trend toward faster psychomotor responses compared to casual players. Regarding temperament, frequent players were characterised by significantly lower self-reported briskness and rhythmicity, while no statistically significant between-group differences were detected for endurance, activity, or emotional reactivity. Crucially, because our data are cross-sectional and the frequent players group comprised exclusively esports students, these differences cannot be interpreted as definitive causal effects or direct consequences of gaming intensity alone.

## 1. Introduction

Video games have undergone a complex evolutionary process, from early games based on simple mechanisms to increasingly complex and enriched environments. Definitions of video games emphasise that they can be played by means of an audiovisual apparatus and may, although not necessarily, be based on a specific storyline [1]. Games achieve their purpose through one of two modes of engagement: rule-based entertainment or interactive fiction, although both forms may also occur simultaneously [2]. In light of the digital game engagement model, games constitute environments based on a dialogue between the player and the computer, creating a cognitive, emotional and kinaesthetic feedback loop. A key element of this experience is also the subjective sense of agency, defined as the player’s desire to exert real control over events in the virtual world. Engagement in games may vary depending on game genre. In three-dimensional worlds (3D), such as first-person shooter games, the player may be engaged through immersive presence within the game world, whereas in more abstract games, such as Tetris, engagement may involve manipulating elements rather than experiencing a sense of being inside the game [3].

Contemporary video game players differ considerably in terms of the amount of time they devote to gaming. Some users play recreationally for a few hours per week, whereas others participate in multi-hour gaming sessions on a daily basis. Research findings indicate that subjectively significant differences in the psychosocial functioning of children and adolescents, as perceived by caregivers, emerge at approximately 4 h and 40 min of daily television viewing and 5 h and 8 min of daily use of digital devices [4]. At the same time, it is emphasised that moderate use of video games may be associated with more favourable indicators of functioning. Turel and Bechara [5] found that adolescents who spent between 1 and 5 h per week playing video games reported significantly less frequent use of psychoactive substances compared with their non-gaming peers. Conversely, excessive engagement in video games is associated with an increased frequency of such use. It should be emphasised, however, that a greater amount of time devoted to gaming does not necessarily translate into poorer psychosocial functioning. As reported by Skripkauskaite et al. [6], for nearly half of adolescents aged 12–18 who played video games for more than four hours per day, gaming still appeared to constitute a healthy and adaptive form of entertainment, whereas measurable difficulties were identified in only approximately 8% of players.

Research conducted among Portuguese adult video game players revealed poor sleep quality. However, the authors emphasised that the participants appeared to maintain a generally healthy lifestyle, characterised by regular physical activity, healthy dietary habits while playing games, and gaming behaviours that did not raise concerns. They also exhibited a healthy emotional profile and good overall well-being [7]. In contrast, another study [8] found that, among adults, an increase in average daily game playing time was associated with a higher prevalence of negative outcomes, including sleep problems, depression, suicidal thoughts, anxiety, obsessive-compulsive symptoms, and alcohol and substance abuse.

As a form of entertainment, computer games may have not only negative consequences, such as addiction, but also positive effects. If games are appropriately adjusted to a child’s age, they may evoke positive emotions, provide knowledge, and teach children how to cope with difficult situations, such as failure. They may also foster perseverance and demonstrate how to engage in competition. In some cases, they may support therapy. Owing to the possibility of selecting the level of difficulty, games allow the player to regulate the degree of emotional arousal. They shape the player’s sense of agency and control and may enhance self-esteem [9]. Li et al. [10] demonstrated that only fifty hours of (action) video game training over a period of nine weeks was sufficient to increase visual sensitivity and contrast sensitivity function among participants. Thus, it was shown that action games may improve the ability to distinguish an object from its background, enhance the ability to locate relevant information among distractors, and facilitate more effective selection of incoming information [9].

To date, analyses of cognitive processes in the context of gaming have focused primarily on the population of esports players, with particular emphasis on reaction time parameters. However, a significant research gap remains regarding cognitive mechanisms among individuals who treat video games purely as a hobby. Empirical attempts to verify the relationship between the actual time devoted to this form of entertainment and such cognitive constructs as focused attention or the ability to manage stress are still relatively rare.

Existing research indicates that individuals who regularly engage in highly dynamic video games achieve significantly shorter reaction times than recreational players, without any loss of accuracy in task performance [11]. The observed improvement in information-processing speed is most likely a direct effect of the optimisation of attentional mechanisms and the strengthening of general cognitive resources [12]. The specific nature of action games requires participants to continuously and selectively monitor the peripheral visual field while effectively ignoring irrelevant distractors, which consequently leads to increased operational efficiency of the brain [13]. Comparative studies have shown that frequent players obtain significantly higher scores in standardised attention tests [14]. Furthermore, experiments conducted by Chandra et al. [15] using the computerised Determination Test (DT) from the Vienna Test System demonstrated that targeted training with action games improves reactive stress tolerance. Nevertheless, these conclusions should be generalised with caution, as the study was conducted on a very small sample of only five participants.

It should also be strongly emphasised that different video game genres do not produce uniform or equally robust changes in visual perception and cognitive control. The cognitive modifications discussed above are most frequently observed in the case of action games, particularly first-person shooters (FPS) [12]. The analysis conducted by Zioga et al. [16] demonstrated that individual video game genres may exert differential effects on players’ cognitive and affective profiles. Role-playing games (RPGs) were positively correlated with both verbal working memory and short-term visuospatial memory, while also showing a negative correlation with empathy. Action games, in turn, were associated with significant improvements in psychomotor speed and selective attention. Puzzle games were positively correlated with visuospatial working memory. Similar observations were made by Lu et al. [17], whose longitudinal research suggests that different video game genres stimulate cognitive functions through distinct neuroplasticity pathways. In particular, action games effectively optimise neural efficiency and generate long-term adaptive effects.

When examining the influence of gaming on cognitive abilities, it is important to take into account the fact that most players, despite having specific genre preferences, rarely limit themselves to one type of gameplay and tend to rotate between different titles. As a result, when analysing this phenomenon from a macrosocial perspective and in terms of its implications for practical life competencies, a holistic approach to gaming activity appears justified, rather than reducing it solely to the isolated effects of a particular game genre [18]. An important area of further exploration is also the role of individual variables, such as temperamental and personality traits, which may determine the degree of engagement in this type of activity and modify its cognitive consequences.

Temperament is a psychological construct that may significantly differentiate the amount of time players devote to video games. In line with the neo-Pavlovian tradition, temperament is assumed to perform a regulatory function, which consists of modifying the stimulatory value of behaviour according to an individual’s temperamental traits [19]. This allows one to assume that video games, as a medium providing strong stimulation, may constitute a form of behavioural regulation. Studies on Internet addiction have indicated that problematic Internet use is determined by a configuration of elevated emotional reactivity and perseverance combined with low levels of briskness and endurance [20]. Similar mechanisms may be observed in relation to the amount of time devoted to video games, where temperamental traits may determine the amount of time spent in front of a computer. Research shows that high negative affectivity and low effortful control, understood as impaired impulse regulation, increase the risk of excessive gaming. Importantly, these traits correlate specifically with addictive behaviours [21]. Additionally, novelty seeking has been identified as a key temperamental trait determining engagement in role-playing games (RPGs); strategy game players have been found to demonstrate higher levels of self-directedness, whereas players who prefer sports games have shown higher reward dependence [22].

Despite growing interest in video games, the relationships between gaming engagement and cognitive functioning in young adults have not yet been clearly established. Most studies focus on children and adolescents. Moreover, relatively few studies have investigated these relationships while taking temperamental traits into account, although such traits may constitute an important factor differentiating both patterns of gaming use and the effectiveness of cognitive processes. Therefore, research employing objective measures of cognitive functioning appears warranted to further examine the relationship between engagement in video games, cognitive functioning and temperamental traits among young adults.

The aim of the present study was to analyse cognitive functioning and temperamental traits among young adults differing in their level of engagement in video gaming. Given the multifaceted nature of these cognitive and psychological constructs, the present study adopts an explicitly exploratory framework rather than a confirmatory one. To guide the reader through the comprehensive battery of analyses, our investigation is structured around three overarching research questions that encompass a total of 31 between-group comparisons:

1. Are there differences between frequent and casual players in cognitive speed and accuracy, as measured by the Reaction Test (RT) and the Determination Test (DT) from the Vienna Test System?
2. Do frequent and casual players exhibit different patterns of sustained attention, as evaluated by the Signal Test (ST) from the Vienna Test System?
3. Do frequent players and casual players differ across the seven distinct dimensions of temperament (e.g., briskness, rhythmicity, sensory sensitivity, emotional reactivity, endurance, activity, and perseverance)?

By addressing these questions individually rather than through a joint omnibus hypothesis, we aim to map out potential behavioural and cognitive variations, establishing a transparent empirical baseline for future targeted replication studies [23].

## 2. Materials and Methods

### 2.1. Participants

The study was conducted at the Regional Centre for Research and Development of the University College in Biała Podlaska. A total of 68 students of esports and physiotherapy from the Józef Piłsudski University of Physical Education in Warsaw, Branch Campus in Biała Podlaska, took part in the study. The research was carried out in a separate, air-conditioned room during the morning hours, under the supervision of the research team. The inclusion criteria were as follows: male, being at least 18 years of age, possessing student status, self-reported good physical and mental health, and providing informed consent to participate in the study. The exclusion criteria included: lack of student status, being under 18 years of age, self-reported health problems that could affect the study results, failure to understand the instructions, failure to complete the study, or providing incomplete responses.

Prior to the study, each participant was informed about the assumptions, aims, course and procedures of the study, as well as about the right to withdraw from participation at any time without giving a reason. All participants provided written, informed and voluntary consent to take part in the research.

The analysis included the results of 67 students from both programmes: esports (n = 40) and physiotherapy (n = 27). One participant was excluded from the study due to an inability to understand the instructions and correctly perform the task.

Participants were divided into two groups based on their daily gaming time: less than 4 h and more than 4 h per day. Although other research indicates that there is no universally accepted classification of players according to gaming intensity [24], we adopted a 4 h cut-off based on previous studies conducted among adults, in which a similar threshold was used to categorise daily gaming time or to characterise intensive gaming [7,8,25,26]. This categorisation was operational in nature and was adopted for the purposes of comparative analyses. The frequent players group (G) consisted of 22 men (n_esport_ = 22; n_physiotherapy_ = 0; age: 22.45 ± 2.40 years) who declared playing video games for more than 4 h per day. Weekly gaming time was originally collected as hours per week and divided by 7 to determine the daily average; the group demonstrated a mean daily gaming time of 6.29 ± 1.62 h (Median = 6.07, IQR = 5.71–7.07, Range = 4.29–11.43). The casual players group consisted of 45 men (n_esport_ = 18; n_physiotherapy_ = 27; age: 21.60 ± 1.96 years) who declared playing video games for less than 4 h per day (a mean daily gaming time of 0.94 ± 0.95 h; Median = 0.71, IQR = 0.14–1.43, Range = 0.00–3.57). No participants in the sample reported a calculated average of exactly 4 h per day.

### 2.2. Study Design

The study began with the collection of basic information from participants concerning the number of hours per week devoted to gaming, followed by the measurement of psychomotor abilities and basic dimensions of temperament. Psychomotor abilities were assessed using tests from the Vienna Test System (VTS; version 8.23.2.59476, SCHUHFRIED GmbH, Mödling, Austria):

(1) The Reaction Test (RT; versions S1, S2 and S3), used to assess the ability to respond to simple stimulus constellations, including simple and choice reactions;
(2) The Signal Test (version S1), which measures long-term focused attention and visual discrimination of a relevant signal in the presence of distracting signals; and
(3) The Determination Test (DT; version S3), used to assess reactive stress tolerance and the ability to respond under complex stimulus conditions.

Before performing each test, participants were familiarised with the nature and course of the upcoming task during an animated instruction phase and an error-sensitive practice phase. All participants completed the procedure in the same order:

(1a) RT/S1—During the test, only one critical stimulus (a yellow circle) was displayed on the monitor screen to which the participant was required to respond. In the test phase, 28 stimuli were presented, each requiring a response. The participant kept one finger on the golden button of the response panel. Upon seeing the yellow circle on the screen, the participant pressed the wide black button also located on the response panel with the same finger as quickly as possible. After pressing the button, the participant placed the finger back on the golden button. The test lasted approximately 4 min.
(1b) RT/S2—During the test, only one critical stimulus was heard (a sound with a frequency of 2000 Hz) to which the participant was required to respond. In the test phase, 28 stimuli were presented, each requiring a response. The participant kept one finger on the golden button of the response panel. Upon hearing the sound, the participant pressed the wide black button located on the response panel with the same finger as quickly as possible. After pressing the button, the participant placed the finger back on the golden button. The test lasted approximately 4 min.
(1c) RT/S3—During the test, visual stimuli (red or yellow circles displayed on the monitor screen), acoustic stimuli (a sound with a frequency of 2000 Hz), and combinations of these signals were presented alternately. The critical combination of stimuli to which the participant was required to respond consisted of a simultaneous visual stimulus (a yellow light) and an acoustic stimulus (a 2000 Hz sound). In the test phase, 48 stimuli were presented, of which 16 required a response. Each time the participant saw a yellow circle on the screen and simultaneously heard the sound, he pressed the wide black button on the response panel with the same finger as quickly as possible. After pressing the button, the participant placed the finger back on the golden button. The test lasted approximately 6 min. The main variables were mean reaction time and mean motor time. Mean reaction time was defined as the interval between stimulus presentation and the initiation of the mechanical motor response, i.e., release of the waiting button. Mean motor time reflected the speed of movement execution. The subsidiary variables were the dispersion of reaction time and the dispersion of motor time, reflecting the variability of reaction time and motor execution time, respectively.
(2) Signal/S1—During the test, white dots were displayed pseudorandomly on a black background on the monitor screen. Some dots disappeared, and others appeared. The critical stimulus configuration to which the participant was required to respond occurred when four directly adjacent dots formed a square. When the participant noticed such an arrangement of dots, he was required to press the green button on the response panel as quickly as possible. The test lasted approximately 15 min. In the Signal Test, the main variable is focused attention, defined as the total number of correct and delayed responses to critical stimuli. Subsidiary variables include median detection time, defined as the median interval between the presentation of a critical stimulus and a correct response, and the number of false alarms, defined as responses made in the absence of a critical stimulus. The number of omitted responses is directly related to focused attention because, given a fixed number of critical stimuli, it represents the complement of the number of correct and delayed responses

After the Signal Test, there was a 5 min break, during which the participant could stand up from the computer desk and rest.

(3) DT/S3—During the test, visual stimuli: circles in five colours (white, yellow, red, green and blue) displayed on the monitor screen and acoustic stimuli: a high-pitched sound with a frequency of 1000 Hz and a low-pitched sound with a frequency of 250 Hz, were presented. The participant’s task was to respond as quickly as possible to all visual and auditory stimuli by pressing the appropriate buttons on the response panel. When a coloured circle appeared, the participant pressed the button of the same colour. Upon hearing a sound, the participant pressed one of two wide rectangular buttons located in the centre of the response panel: the upper grey button for the high-pitched sound and the lower black button for the low-pitched sound. The buttons assigned to the five colours were arranged on the response panel in such a way that the participant could use both hands. The next stimulus appeared only after the correct response had been provided; the speed of stimulus presentation is determined by the respondent. The test lasted approximately 8 min. In the DT Test Form S3, the main variable is median reaction time in action mode. The subsidiary variable correct reflects the respondent’s ability to react quickly and appropriately during a series of simple reaction tasks.

Individual differences were assessed in terms of the basic dimensions of temperament. Participants completed the questionnaire at a convenient time, after performing the VTS tests. For this purpose, the standardised Polish version of the Formal Characteristics of Behaviour—Temperament Inventory was used (Formalna Charakterystyka Zachowania—Kwestionariusz Temperamentu, revised version FCZ-KT(R) [27]. The instrument demonstrates high internal consistency as well as good temporal stability. In the original validation study, Cronbach’s alpha coefficients for the individual scales range from 0.73 to 0.88, indicating high measurement reliability. The instrument has also demonstrated high validity; validation studies examined the relationships between temperament and other personality dimensions, cognitive characteristics, and intelligence.

The questionnaire consists of 100 statements to which participants responded using a 4-point scale. The FCZ-KT(R) contains seven content scales. Three of them are time-defined dimensions: briskness, perseverance and rhythmicity, while the remaining four are energy-defined dimensions: sensory sensitivity, endurance, emotional reactivity and activity.

1. Briskness—The tendency to maintain a high pace of performed activities, energetic action and appropriate adaptation to the environment.
2. Perseverance—A high level indicates a tendency to analyse events and previous decisions in detail, especially those associated with failures and negative emotions. It also refers to the tendency to repeat behaviours and emotional reactions despite the cessation of the stimulus that evoked them.
3. Sensory sensitivity—The tendency to react strongly to sensory stimuli of low stimulatory value; a high level indicates stronger perception and discrimination of sensory stimuli.
4. Endurance—The ability to respond adequately in situations requiring prolonged and highly stimulating activity; the ability to remain active under unfavourable conditions; and the capacity for prolonged work despite pain, noise, fatigue and similar factors.
5. Emotional reactivity—The ease with which emotions are aroused even under weak stimulation, sensitivity to emotional stimuli, low emotional resilience and a tendency for performance to decrease in stressful situations.
6. Activity—The tendency to undertake numerous activities, low tolerance of monotony, and the tendency to regulate the level of arousal by deliberately providing the body with stimulation through diverse activities.
7. Rhythmicity—The tendency to perform everyday activities regularly and rhythmically and to maintain a regular lifestyle [27].

### 2.3. Statistical Analysis

Statistical analysis was performed using the Jamovi statistical package (version 2.7; jamovi.org). An a priori power analysis was conducted using G*Power software (version 3.1.9.7) for a two-tailed Wilcoxon Mann–Whitney test. Assuming a medium-to-large effect size (d = 0.70) and a significance level (α = 0.05), a total sample size of 70 observations is required to achieve the standard statistical power of 0.80.

In the first step, the normality of the distribution of the variables was assessed using the Shapiro–Wilk test. The results showed that, for most variables, the distribution significantly deviated from normality (*p* < 0.05). Therefore, the non-parametric Mann–Whitney U test was used to examine between-group differences. Unadjusted *p*-values were retained for all statistical comparisons, and the alpha level was maintained at 0.05. In line with the methodological framework described by García-Pérez [23], corrections for multiple testing (such as Bonferroni or FDR adjustments) were deliberately omitted. The parameters under investigation—including the distinct temperament traits from the FCZ-KT(R), the psychomotor metrics from the Vienna Test System (RT S1, RT S2, RT S3), and the cognitive indicators from the SIGNAL and DT tests—represent separate, autonomous behavioural and neurocognitive dimensions. Furthermore, multiple indicators evaluated within the same subtest often involve composite indices alongside their direct constituent metrics (e.g., the focused attention indicator in the SIGNAL test, which is structurally dependent on the correct and delayed responses). Because these variables are mathematically dependent and nested, applying multiplicity adjustments would result in severe over-correction, excessively inflating Type-II error rates and compromising statistical power for individual, substantive conclusions.

The rank-biserial correlation coefficient (rbs) was used as a measure of effect size for between-group differences. The magnitude of the rank-biserial correlation coefficient (rbs) was systematically interpreted based on the non-parametric benchmarks proposed by Vargha and Delaney [28], where effect sizes are classified as negligible (|rbs| < 0.11), small (0.11 ≤ |rbs| < 0.28), medium (0.28 ≤ |rbs| < 0.43), and large (|rbs| ≥0.43).

Descriptive statistics were presented as medians, that is, the 50th percentile, and interquartile ranges, that is, the 25th and 75th percentiles. Additionally, for informative purposes, means and standard deviations (SD) were also included in the tables.

## 3. Results

### 3.1. Assessment of Reaction Time

The results of the analysis using the Mann–Whitney U test did not indicate any statistically significant differences (*p* > 0.05) in the RT test scores (versions S1, S2 and S3) between frequent players and casual players (Table 1).

### 3.2. Assessment of Long-Term Focused Attention

The analysis of the Signal Test results (Table 2) revealed statistically significant differences between the study groups in the following variables: number of omitted reactions (U = 295; *p* = 0.007, rbs = 0.41); number of correct reactions (U = 284, *p* = 0.005, rbs = −0.43), focused attention (U = 295, *p* = 0.007, rbs = −0.41) and total results (U = 285, *p* = 0.005, rbs = −0.42). Frequent players omitted stimuli less frequently, provided a greater number of correct responses, and achieved higher scores for focused attention and total results.

### 3.3. Assessment of Reactive Stress Tolerance and the Associated Ability to React

The analysis of the Determination Test results did not indicate any statistically significant differences (*p* > 0.05) between the groups (Table 3).

### 3.4. Assessment of Temperamental Traits

For the FCZ-KT(R) questionnaire results, two statistically significant differences were found between the groups: briskness (U = 336, *p* = 0.034, rbs = 0.32) and rhythmicity (U = 323, *p* = 0.021, rbs = 0.35). For both variables, frequent players obtained lower scores (Table 4).

## 4. Discussion

The aim of the study was to analyse cognitive functioning and temperamental traits among young adults differing in their level of engagement in video gaming. In response to our first two research questions, the results indicate a nuanced cognitive profile: frequent players demonstrated a robust advantage in sustained attention parameters from the Signal Test, alongside a marginal, descriptive trend toward faster psychomotor responses in the Reaction Test. Regarding the third research question, the data point to a specific configuration of temperamental traits, characterised by significantly lower self-reported briskness and rhythmicity among frequent players compared to casual players, while no statistically significant between-group differences were detected for endurance, activity, or emotional reactivity. Within this exploratory framework, these findings suggest that unique cognitive efficiencies and temperamental patterns co-occur in a tentative configuration rather than reflecting an established causal process.

In our study, the results obtained in the RT point toward a statistical trend with a medium effect size, which cautiously suggests that frequent players may exhibit faster reaction time than casual players. The Signal Test showed that frequent players omitted stimuli less frequently, demonstrated higher accuracy in responding to specific signals, and obtained a significantly higher focused attention score compared with the casual players. Crucially, these differences were supported by medium-to-large effect sizes, with the accuracy of responses demonstrating a large effect, while the omission rates and focused attention scores reflected prominent medium effects. The overall Signal Test further confirmed this statistically significant advantage of the frequent players group. These results may be interpreted twofold, though both perspectives require careful consideration. First, they may be viewed as tentative patterns aligning with the cognitive plasticity hypothesis, according to which the video game environment might stimulate and enhance users’ cognitive functions. Second, they may reflect a potential selection effect: frequent players may have initially possessed a slightly higher level of cognitive abilities, which could have facilitated success in games and consequently motivated them to invest a substantial amount of time in this activity. In relation to the first interpretation, the literature offers a suggestive theoretical and empirical framework regarding the stimulating potential of video games. For instance, the meta-analysis by Bediou et al. [12] indicates that regular gameplay, particularly action video game play, causally improves cognitive performance, producing the strongest effects in the domains of attention, perception and spatial abilities. It should be emphasised, however, that these causal relationships apply to a specific genre, namely action games. These conclusions have also been subject to substantial criticism in the literature. Meta-analyses focusing on the absence of far cognitive transfer [29] and large-scale cross-sectional studies suggest that general engagement in games with diverse genre characteristics, such as RPGs or turn-based games, does not translate unequivocally into improved basic cognitive functions [30].

In the present study, participants reported playing a variety of game titles, including RPG, FPS, RTS and turn-based games. This fact requires the consideration of an alternative explanation. From a motivational perspective, such as Self-Determination Theory (SDT) [31,32], individuals with higher initial cognitive abilities might acquire complex game mechanics more efficiently. This could enhance their subjective sense of competence and autonomy, subsequently fostering longer gaming engagement. While it is plausible that a pre-existing cognitive profile promotes self-selection into intensive gaming, our cross-sectional design cannot establish causal precedence.

The analysis of the cognitive test results (significant differences in the Signal Test and a numerically shorter reaction time for which the between-group comparison did not reach statistical significance in the S3 version of the RT) when considered together with the temperamental profile of the examined frequent players, reveals an interesting paradox. From the perspective of Strelau’s Regulatory Theory of Temperament, the lower level of briskness observed among frequent players, defined as the ability to react quickly and switch easily between activities, should theoretically align with longer reaction time and lower flexibility in attention-based tests. In the present sample, however, a descriptive divergence was observed: despite lower self-reported briskness, frequent players demonstrated robust efficiency in sustained attention and a descriptive trend toward faster psychomotor responses.

This phenomenon could tentatively be explained by a potential synergistic effect of self-selection and specific cognitive training. Individuals with lower self-reported briskness initially select the video game environment because it offers a highly structured and safe space for activity. Subsequently, through prolonged and frequent playing experience, they might develop enhanced procedural automatization and so-called motor memory, or muscle memory [33,34]. Lee et al. [35] indicated that success in competitive games, such as League of Legends or Counter-Strike, is largely dependent on repetitive patterns and motor habits. Within this framework, a player with lower self-reported briskness may not necessarily need to constantly adapt to entirely new stimuli, but could instead activate highly practised, predefined responses. Such specific training could potentially facilitate a behavioural compensation for tasks whose structure shares common characteristics with gaming mechanics, as might be the case with the Vienna Test System parameters used in the present study. This tentative interpretation appears to align with the perspective proposed by Sala et al. [29], who argue that the potential cognitive benefits of gaming are mainly limited to so-called near transfer, that is, a situation in which the test task directly reflects cognitive operations practised during gameplay.

One potentially important finding of the present study suggests that individuals with lower self-reported briskness and rhythmicity might find infrequent playing in an environment that is compatible with their individual style of functioning, a pattern that was supported by a medium effect size. Low briskness predisposes individuals to deep information processing, while at the same time making everyday, multi-threaded reality, which requires constant multitasking and adaptation to unpredictable stimuli, highly energy-demanding and disruptive [19]. Although video games are commonly perceived as dynamic, they are in fact structured environments with a closed catalogue of rules and stimuli [13]. Even the most unpredictable esports game happens within the rigid framework of a defined map and mechanics. This predictability might effectively “close” the stimulus space within the screen and speakers, which could potentially help individuals with lower self-reported briskness achieve a more stable state of deep concentration, conceptually related to the phenomenon of flow. While individuals with high briskness may be more easily drawn toward new sensations, frequent players with lower self-reported briskness may potentially demonstrate greater persistence within a stable, screen-based activity [36]. On the other hand, according to Nęcka et al. [37], briskness is a significant positive predictor of self-control, suggesting that higher briskness may be associated with an increase in self-controlled behaviours. It may therefore be hypothesised that lower self-reported briskness among frequent players could potentially affect their capacity to adapt rapidly to the environment or shift smoothly from their current behaviour (i.e., playing video games) to another activity. This underlying temperamental characteristic might, in turn, represent a factor that favours multi-hour gaming sessions. Similar conceptual patterns can be drawn from the study by Dudziak and Sitarczyk [20], in which the group at high risk of Internet addiction obtained significantly lower scores in briskness than the group at low risk of Internet addiction. However, because our data are cross-sectional and did not measure addictive behaviours directly, these directional relationships must remain speculative until verified by longitudinal research.

Similarly, the lower self-reported rhythmicity among frequent players—supported by a medium effect size—suggests a tentative hypothesis regarding lifestyle compatibility. Rather than assuming that gaming solely disrupts circadian regulation, individuals with a naturally looser structure of biological rhythms might self-select into gaming as a flexible environment that lacks rigid temporal constraints [38]. Furthermore, the absence of significant differences in Endurance, Activity, and Emotional Reactivity challenges traditional reductive models that equate intensive gaming with pathological escapism or psychological vulnerability [39]. Instead, frequent gaming within this exploratory framework appears to represent a functional search for an optimal level of stimulation tailored to the players’ personal pace and structural flexibility.

### Limitation

The present study has several important limitations that should be taken into account when interpreting the obtained results. First, the study was conducted on a relatively small sample, which limits the possibility of generalising the findings to the wider population of frequent players. Another limitation of the present study concerns a potential selection bias and the presence of a confounding variable related to the participants’ academic fields. Since the frequent players group consisted exclusively of esports students, while the casual players group predominantly comprised physiotherapy students alongside a smaller subgroup of esports students, the observed between-group differences cannot be interpreted solely as a direct consequence of gaming intensity. The distinct fields of study might represent a systematic confounder; for instance, the specific curriculum, career orientations, or pre-existing self-selection into academic programs like physiotherapy versus esports may independently align with particular temperamental traits and baseline cognitive speeds. Consequently, these demographic characteristics further emphasise the exploratory and tentative nature of our findings, and future studies should recruit more educationally homogeneous cohorts to isolate the independent effects of media engagement. Second, the study included only men, which makes it impossible to assess potential sex-related differences. Third, an important limitation is the lack of detailed information regarding the specific games played by the participants. Moreover, the study did not include a control group consisting of individuals who do not play video games. The absence of such a comparison group makes it difficult to determine unequivocally whether the observed results are related to gaming experience or whether they stem from other individual characteristics of the participants. Another important limitation is the lack of control over participants’ level of fatigue at the time of testing. No information was collected regarding the quality of sleep on the previous night, the participants’ level of rest, or their current well-being, all of which may affect performance on cognitive tasks. Future studies should include a larger sample comprising both women and men, precisely define the type of games played, take into account the roles performed by players within specific games, control for participants’ level of fatigue, and include an appropriate control group.

## 5. Conclusions

The present exploratory study suggests a nuanced relationship between media engagement, specific cognitive performance, and temperamental traits. Frequent players demonstrated a robust advantage in parameters of sustained attention, alongside a marginal descriptive trend toward faster psychomotor responses compared to casual players. Regarding temperament, frequent players were characterised by significantly lower self-reported briskness and rhythmicity, while no statistically significant between-group differences were detected for endurance, activity, or emotional reactivity.

Crucially, because our data are cross-sectional and the frequent players group comprised exclusively esports students, these differences cannot be interpreted as definitive causal effects or direct consequences of gaming intensity alone. Instead, these outcomes could tentatively reflect a broader selection mechanism, where individuals with unique pre-existing cognitive profiles and specific temperamental styles self-select into compatible digital environments and related educational paths. Future longitudinal research utilising educationally homogeneous cohorts is required to firmly isolate the independent effects of video game engagement from broader lifestyle and institutional confounders.

## Figures and Tables

**Table 1:** U Mann–Whitney results and descriptive statistics for Reaction Test (RT) among frequent players group (F) and casual players group (C).

VST Test	Group	Mean	SD	Percentiles				U Mann–Whitney	
				25th	50th	75th	U	*p*	rbs
RT/S1									
Motor time [ms]	G	107.41	28.64	85.00	117.50	126.50	468	0.723	−0.05
	C	106.49	26.00	91.00	102.00	116.00			
Reaction time [ms]	G	238.96	22.67	221.80	239.50	248.50	455	0.598	0.08
	C	242.67	27.99	218.00	245.00	264.00			
Motor time dispersion [ms]	G	15.50	5.15	12.30	15.00	17.80	432	0.400	0.13
	C	17.31	7.19	12.00	17.00	21.00			
Reaction time dispersion [ms]	G	24.46	7.79	19.00	23.50	26.00	417	0.300	0.16
	C	25.09	5.89	21.00	25.00	28.00			
RT/S2									
Motor time [ms]	G	106.14	23.74	94.00	108.00	120.00	401	0.212	−0.19
	C	102.29	21.47	88.00	99.00	113.00			
Reaction time [ms]	G	209.96	34.47	191.00	201.50	219.80	439	0.459	0.11
	C	211.27	25.24	193.00	206.00	229.00			
Motor time dispersion [ms]	G	12.46	4.69	10.00	10.50	13.80	387	0.149	0.22
	C	14.31	5.93	10.00	14.00	17.00			
Reaction time dispersion [ms]	G	23.59	8.52	18.00	22.00	26.00	471	0.753	0.05
	C	23.62	7.06	19.00	24.00	26.00			
RT/S3									
Motor time [ms]	G	114.14	31.02	99.30	111.50	121.80	428	0.371	−0.14
	C	107.91	24.56	93.00	106.00	117.00			
Reaction time [ms]	G	318.59	45.71	285.50	313.50	334.80	356	0.064	0.28
	C	342.38	54.85	306.00	334.00	354.00			
Motor time dispersion [ms]	G	15.32	7.09	11.00	13.00	17.80	483	0.878	−0.02
	C	14.60	5.26	10.00	13.00	18.00			
Reaction time dispersion [ms]	G	50.23	13.73	41.30	49.50	57.50	467	0.708	−0.06
	C	49.91	14.21	41.00	48.00	54.00			

**Table 2:** U Mann–Whitney results and descriptive statistics for the Signal test among the frequent players group (F) and the casual players group (C).

VST Test Signal/S1	Group	Mean	SD	Percentiles			U Mann–Whitney		
				25th	50th	75th	U	*p*	rbs
Focused attention [n]	G	53.73	5.19	50.50	55.00	58.00	295	0.007	−0.41
	C	51.36	3.64	49.50	52.00	54.00			
Correct reactions [n]	G	53.32	5.21	51.30	55.00	57.00	284	0.005	−0.43
	C	50.84	3.74	49.00	51.00	54.00			
Delayed reactions [n]	G	0.41	0.67	0.00	0.00	1.00	451	0.500	0.09
	C	0.51	0.70	0.00	0.00	1.00			
Omitted reactions [n]	G	6.27	5.19	2.25	5.00	8.75	295	0.007	0.41
	C	8.64	3.64	6.00	8.00	10.00			
Total [n]	G	46.18	10.18	37.50	49.50	54.00	285	0.005	−0.42
	C	40.62	7.63	37.50	42.00	45.50			
Median detection time [s]	G	0.72	0.10	0.65	0.72	0.77	480	0.841	0.03
	C	0.73	0.08	0.67	0.72	0.78			
Average detection time [s]	G	0.87	0.21	0.70	0.84	0.97	396	0.188	0.20
	C	0.94	0.18	0.81	0.88	1.04			

Total = focused attention—number of omitted reactions—number of false reactions.

**Table 3:** U Mann–Whitney results and descriptive statistics for the Determination Test (DT) among the frequent players group (F) and the players group (C).

VST Test DT/S3	Group	Mean	SD	Percentiles			U Mann–Whitney		
				25th	50th	75th	U	*p*	rbs
Median reaction time [s]	G	0.67	0.05	0.63	0.67	0.70	474	0.779	−0.04
	C	0.66	0.05	0.63	0.66	0.70			
Correct reaction [n]	G	328.27	20.15	311.75	328.50	343.75	467	0.713	0.06
	C	331.31	23.37	316.00	330.00	344.00			
Incorrect reaction [n]	G	18.91	11.48	13.25	17.50	23.75	462	0.664	−0.07
	C	20.13	17.86	9.00	15.00	26.00			
Number of reactions [n]	G	347.18	25.22	333.25	346.00	356.00	462	0.659	0.07
	C	351.44	30.42	329.00	348.00	361.00			

**Table 4:** U Mann–Whitney results and descriptive statistics for temperamental traits (FCZ-KT (R)).

Scales FCZ-KT(R)	Group	Mean	SD	Percentiles			U Mann–Whitney		
				25th	50th	75th	U	*p*	rbs
Briskness	G	44.91	6.15	41.75	44.00	48.25	336	0.034	0.32
	C	47.31	4.24	45.00	48.00	50.00			
Activity	G	36.45	6.19	32.75	36.00	39.25	376	0.111	0.24
	C	39.71	7.31	33.50	39.00	44.00			
Emotional reactivity	G	35.86	8.00	29.75	38.00	41.25	451	0.561	−0.09
	C	35.00	6.21	30.00	35.00	38.50			
Endurance	G	38.77	7.39	33.50	38.50	45.00	435	0.422	−0.12
	C	36.93	7.64	30.50	38.00	42.50			
Sensory sensitivity	G	43.09	4.86	39.50	43.00	47.00	457	0.616	0.08
	C	43.36	4.48	39.50	44.00	47.00			
Perseverance	G	43.77	4.97	39.00	44.00	47.25	453	0.574	−0.09
	C	43.09	6.28	39.00	43.00	47.00			
Rhythmicity	G	17.27	4.71	14.75	17.00	19.25	323	0.021	0.35
	C	20.00	5.25	17.00	19.00	22.00			

## Data Availability

The original contributions presented in this study are included in the article. Further inquiries can be directed to the corresponding author.
